# Assessing Long-Term Test-Retest Reliability of the CPT-IP in Schizophrenia

**DOI:** 10.1371/journal.pone.0084780

**Published:** 2014-01-08

**Authors:** Eric Hahn, Andrea Vollath, Tam T. M. Ta, Constanze Hahn, Linn K. Kuehl, Michael Dettling, Andres H. Neuhaus

**Affiliations:** 1 Department of Psychiatry and Psychotherapy, Charité-University Medicine, Campus Benjamin Franklin, Berlin, Germany; 2 Department of Biopsychology, Ruhr University, Bochum, Germany; Hangzhou Normal University, China

## Abstract

**Background:**

The Continuous Performance Test-Identical Pairs version (CPT-IP) is a well-established measure of sustained attention, and its more challenging versions are particularly suited to detect subtle processing deficits in patients with schizophrenia. However, while there are few longitudinal samples for the CPT-IP, no study has addressed stability for more than two month in patients with schizophrenia. Assessing long-term test-retest reliability of the CPT-IP would facilitate the ability of clinicians to draw conclusions from studies involving interventions as long term cognitive or pharmacological treatments. The present study assessed 12 month test-retest reliability for the two most challenging versions of CPT-IP (4-digit and shapes) in a matched sample of clinically stable schizophrenia outpatients and healthy controls.

**Methods:**

Fifty clinically stable schizophrenia outpatients and 50 healthy controls were assessed with the CPT-IP for the 4-digit and shape conditions. From these, 40 patients and 47 controls were reassessed with an average interval of 12.3 months between test sessions. Test-retest reliability was analyzed with Pearson correlations and results were compared with previous data involving healthy controls and short-term studies in patients with schizophrenia.

**Results:**

Especially d’ and hit rate discriminated well between patients with schizophrenia and healthy controls for both CPT-IP conditions and at both test sessions. Healthy controls demonstrated sufficient long term test-retest correlations of d’, hit rate and reaction time for both the 4-digit and shape conditions. However, in schizophrenia patients, long-term reliability correlations were at best moderate for d’ and hit rate only.

**Conclusions:**

The current study provides further evidence that d’ and hit rate yield consistent cross-sectional discrimination sensitivity. At best moderate long-term test-retest reliability of d’ in schizophrenia outpatients may be not sufficient for practical use of this measure in long term clinical trials.

## Introduction

Attention deficits are well established symptoms of patients with schizophrenia that are associated with functional outcome in longitudinal studies and are considered valid predictors of impaired cognitive functions in schizophrenia [1], [2]. Today the Continuous Performance Test (CPT) has emerged as the standard measure of deficits in sustained attention and such deficits are among the most robust cognitive measures that may predict the onset of schizophrenia spectrum disorders in at-risk individuals [3] and may differentiate between schizophrenia, depression and bipolar disorder [4], [5].

The Identical Pairs CPT (CPT-IP) [4], [6] especially in its more challenging versions was specifically designed to detect subtle processing deficits of sustained attention at early stages of schizophrenia and in at-risk individuals. Here subjects have to respond to a second stimulus in any pair of identical stimuli that may include 2, 3 or 4 digits and shapes, which activate the verbal and spatial attentional neuronal systems [7]. Especially in its more challenging versions CPT-IP performance deficits were also found in prodromal stages of schizophrenia, in first episode neuroleptic-naive schizophrenic patients [8], in individuals within the schizophrenia spectrum, including schizotypal personality traits [9]–[11], and in asymptomatic first-degree relatives of patients with schizophrenia [12].

In contrast to strong evidence for CPT-IP task validity, data on its long-term reliability in independent samples is scarce. While good short and long-term test-retest reliability was assessed for healthy controls [6], [13], studies investigating this important issue in schizophrenia have been conducted only for short-term, i.e. 2-week [14] or 4-week intervals [15]. A recent study aimed at standardization of the CPT-IP in schizophrenia research assessed the reliability in 43 patients with schizophrenia and 40 healthy controls in four different versions of the CPT-IP at three test sessions over five weeks. In this study, all four versions of the CPT-IP demonstrated good short-term reliabilities and discrimination sensitivity [16]. Results of that study also indicated that the 4-digit CPT and the shapes CPT were the most challenging versions for schizophrenia patients [16].

Overall, while in healthy individuals relevant CPT-IP measures appeared to be psychometrically reliable for short and long-term intervals and while well designed studies demonstrated good short-term reliability in schizophrenia, studies assessing long-term reliability of CPT-IP measures in patients with schizophrenia are to date still lacking. In an attempt to fill this methodological gap, the current study aimed at investigating test-retest reliability for the most challenging versions (4-digit and shapes) of the CPT-IP in schizophrenia for a between test sessions interval of 12 month. Such long term reliability data of this standard measure of sustained attention can be useful when interpreting possible results of longitudinal effects of interventional studies aimed at improving attentional deficits in patients with schizophrenia.

## Methods

### Participants

All participants agreed to repeat the both CPT-IP tests approximately 12 months after the first session and gave written informed consent before participating in the study. The study protocol was approved by the ethics committee of the University Hospital Campus Benjamin Franklin of the Charité University Medicine Berlin, and the study was conducted in accordance with the Declaration of Helsinki. All participants were right-handed [17], reported normal or corrected-to-normal vision, and were of European descent.

### Patients with Schizophrenia

Fifty patients (20 female) meeting DSM-IV criteria for schizophrenia were initially enrolled at baseline. Patients met the following inclusion criteria: age 18–65; no major change in antipsychotic medications defined as switching to another antipsychotic class and no psychiatric hospitalization for at least six weeks prior to entering the study. Patients were excluded from the study when meeting any of the following criteria: psychiatric inpatient status between test sessions, history of severe medical or neurological disorder, substance abuse/dependence other than nicotine as evidenced by urinary drug screening, history of electroconvulsive therapy, current extra-pyramidal symptoms, current treatment with mood stabilizers; antidepressants or anticholinergic agents and benzodiazepine co-medication within six weeks prior to testing. Forty patients (15 female) remained clinical stable within one year, had no major change of antipsychotic medication and accepted reassessment (80% retention rate). Between both test sessions, all patients had monthly psychiatric appointments to ensure continuous clinical monitoring during the study. All patients were recruited from the outpatient unit of the Department of Psychiatry and Psychotherapy, Campus Benjamin Franklin, Charité-University Medicine Berlin in Germany.

### Psychopathology and Medications

During the initial screening and final assessment, patients were administered the Positive and Negative Symptom Scale (PANSS) as a measure of psychopathology severity [18]. We tried to assess test-retest reliability of CPT-IP measures independent of changes in psychopathology between test sessions and therefore included only clinically stable patients who had a severity rating of moderate or less (≤4) on selected PANSS positive scale items (conceptional disorganization, hallucinations, hyperactivity and hostility) that most likely could interfere understanding or valid execution of the CPT-IP testing [19]. PANSS ratings were performed by author EH at both days of CPT-IP testing. Symptom levels were mild, as reflected by a PANSS total score of 62.9 at test and 58.4 at retest sessions [19]. None of the patients had a PANSS score >4 (moderate) for any single item on the positive subscale. Mean PANSS scores between test sessions did not differ significantly, as measured by the positive (13.33±4.6 vs. 13.18±3.5; T_39_ = 0.179; p = .86), negative (16.70±4.5 and 15.35±4.9; T_39_ = 1.546; p = .13) and general subscales (31.90±7.1 and 29.88±7.5; T_39_ = 1.381; p = .18). All patients received oral second generation antipsychotic medication: amisulpride (N = 6), aripiprazole (N = 9), clozapine (N = 11), olanzapine (N = 7), paliperidone (N = 2), quetiapine (N = 5), risperidone (N = 14), ziprasidone (N = 1). Twenty-five patients received a second generation antipsychotic mono-therapy, and 15 patients received a second generation antipsychotic combination therapy. Calculation of chlorpromazine (CPZ) equivalents was performed following the suggestion of Andreasen et al. [20]. CPZ equivalents of oral second generation antipsychotic medication between baseline and retest session did not differ significantly (557.45±347.11 vs. 542.43±299.15; T_39_ = 0.527; p = .60). Additionally, CPZ equivalents were highly correlated (r = .855; p<.001), and none of the patients was switched to a different antipsychotic medication class between test sessions, thus further ensuring equivalent psychopharmacological conditions within the patient group over time.

Demographic and clinical data are summarized in **Table 1**.

**Table 1:** Summary of demographic and clinical data at baseline and retest (mean ± SD).

	Baseline Schizophrenia	Baseline Controls	Retest Schizophrenia	*p*
N (female/male)	40 (15/25)	47 (19/28)	–	n.s.^a^
Age [years]	34.4±10.2	33.9±9.5	–	n.s.^b^
Education [years]	13.7±1.8	14.2±1.8	–	n.s.^b^
Smokers/non-smokers	25/15	28/19	–	n.s.^a^
Nicotine [pack years]	7.48±9.0	6.51±8.1	–	n.s.^b^
Test interval [days]	377.7±17.7	372.5±7.6	–	n.s.^b^
DOI [months]	89.74±68.6	–	–	–
N episodes	3.07±1.9	–	–	–
PANSS positive scale	13.33±4.6	–	13.18±3.5	n.s.^c^
PANSS negative scale	16.70±4.5	–	15.35±4.9	n.s.^c^
PANSS general scale	31.90±7.1	–	29.88±7.5	n.s.^c^
CPZ equivalents [mg/d]	557.45±347.1	–	542.43±299.2	n.s.^c^

DOI, duration of illness; PANSS, Positive and Negative Syndrome Scale;

CPZ, chlorpromazine.

^a^χ^2^ test;

b *t*-test for independent samples,

c *t*-test for paired samples.

### Healthy Controls

Fifty healthy control participants (20 female) were recruited via advertisements in a local newspaper and on the department’s homepage. Controls were initially matched for age, sex, smoking status and education years. Forty-seven control participants (19 female) were reassessed at follow-up (94% retention rate) and were entered into analysis. Three control participants had moved to another city and thus declined reassessment. Control participants were excluded if they met any of the following criteria: history of psychiatric axis I disorder according to DSM-IV, history of sustained abuse of alcohol or drugs other than tobacco smoking, severe medical or neurological condition and any psychopharmacological treatment in the past. A first-degree family history of psychiatric axis I disorder likewise led to exclusion from the study. All control participants were examined by a certified psychiatrist prior to inclusion using the Mini-International Neuropsychiatric Interview for DSM-IV [21].

### Continuous Performance Test–Identical Pairs Version (CPT-IP)

Sustained attention was measured using the two most challenging versions of the CPT-IP developed by Cornblatt et al. [6]. Both versions included 300 trials for the 4-digit and 300 trials for the shapes condition. All stimuli were presented in a distance of 60 cm on a 19-inch TFT monitor located in a sound- and light-attenuated testing room. Each stimulus was presented for 50 ms, followed by an inter-stimulus interval of 950 ms, resulting in a total trial time of 1000 ms. Subjects were asked to respond as fast and as accurately as possible (via a finger lift from a response key of their dominant right hand) as soon as two consecutive stimuli looked identical [3]. Following 50 practice trials with three-digit numbers and shapes, 2×300 test trials were administered, divided into two successive blocks for both conditions in a counterbalanced order. In each condition, 20% target pairs and an equal percentage of “false alarm” pairs – i.e. catch trials – were presented. Another 60% of randomly presented stimuli served as organized fillers. Participants did not have any information regarding the proportions of trial stimuli.

Outcome measures were calculated for the digits and shapes conditions separately. The primary outcome measure, d’ assesses the ability of the participant to discriminate between signal and noise. Secondary outcome measures were hit rate (percent correct hits), mean reaction time (RT) for correct hits and calculated measures for the response bias (ln ß) and (log random) as measure of responses to irrelevant stimuli.

### Statistical Analysis

Statistical calculations were conducted using SPSS for Windows 19.0 (IBM, Armonk, NY, US). All tests were performed as two-tailed tests with an alpha level set at *p*<.05. Demographic and clinical data were analyzed with χ^2^ tests, t-tests for independent and for paired samples, as appropriate. Primary outcome measures, i.e. CPT-IP variables d’, hit-rate, mean reaction time, ln ß, and log random for 4-digit and shape stimuli, were submitted to a repeated measures analysis of variance (ANOVA). Time was entered as a within-subject factor and diagnostic group was entered a between-subject factor, thus resulting in a 2×2 ANOVA design, which was applied to every primary outcome measure separately. As this approach results in ten separate repeated measures ANOVAS, a Bonferroni correction was applied. To compare our results with those of previous studies, longitudinal data, i.e. test-retest reliability proper, were psychometrically analyzed computing Pearson’s r correlation coefficients (Cook and Beckman, 2006). For both reliability measures, we initially defined the thresholds for reliability in our study protocol following Altman who considered r<0.4 as poor, r = 0.4–0.6 as moderate, r = 61–.8 as good, and r>.81–1.0 as very good reliability [22].

## Results

The mean interval between test and retest was 12.3 months (range 11.8–13.7 months) for both groups. Mean intervals between test sessions did not differ significantly between healthy controls (372.5±7.6 days) and patients with schizophrenia (377.7±17.2 days).

### Omnibus ANOVAs

Repeated measures ANOVAs demonstrated significant main effects of ‘diagnostic group’ for the outcome variables d’ for both digits (F(1,85) = 23.87; p<.001) and shapes (F(1,85) = 32.18; p<.001); hit rate for both digits (F(1,85) = 28.65; p<.001) and shapes (F(1,85) = 31.71; p<.001); and log random for digits only (F(1,85) = 20.41; p<.001). No significant main effects of ‘time’ or significant interactions of ‘time*diagnostic group’ for any outcome variable were found. Complete raw data is available on request by the corresponding author.

### Longitudinal Data

Test-retest reliability data are presented stratified by group and condition in **Table 2**. Pearson’s r correlations indicated that d’ (4-digit: r = .812; p<.001; shapes: r = .729; p<.001); hit rate (4-digit: r = .707; p<.001; shapes: r = .603; p<.001); and mean RT (4-digit: r = .739; p<.001; shapes: r = .732; p<.001) showed good long-term retest reliability in healthy controls. In patients with schizophrenia, d’ (4-digit: r = .502; p<.001; shapes: r = .529; p<.001) and hit rate (4-digit: r = .459; p<.001; shapes: r = .410; p<.01) were only moderately correlated.

**Table 2:** Test-retest correlations of CPT-IP performance measures (mean ± SD).

Condition	CPT-IP measures	Baseline Controls	Retest Controls	Pearson r
**CPT-4-digit**	d’	1.73±0.9	1.78±0.8	0.812***
	hit rate (%)	75.60±18.0	76.98±18.5	0.707***
	RT hits [ms]	532.25±61.5	537.06±65.9	0.739***
	ln ß	-.10±0.8	-.09±0.7	0.370**
	log random	0.34±0.5	0.16±0.3	0.286
**CPT-shapes**	d’	1.96±0.7	2.28±0.6	0.729***
	hit rate (%)	76.23±16.3	81.15±14.7	0.603***
	RT hits [ms]	512.49±65.0	524.15±68.5	0.732***
	ln ß	0.09±0.8	0.17±1.1	0.636***
	log random	0.53±0.6	0.64±0.5	0.194
**Condition**	**CPT-IP measures**	**Baseline Schizophrenia**	**Retest Schizophrenia**	**Pearson r**
**CPT-4-digit**	d’	1.00±0.7	1.10±0.6	0.502***
	hit rate (%)	52.75±23.3	57.45±23.7	0.459***
	RT hits [ms]	550.41±80.4	541.89±116.6	0.281
	ln ß	0.30±0.7	0.23±0.6	0.377*
	log random	0.80±0.7	0.57±0.6	0.253
**CPT-shapes**	d’	1.29±0.9	1.31±0.9	0.529***
	hit rate (%)	67.35±24.5	67.12±26.4	0.410**
	RT hits [ms]	525.53±78.1	528.55±118.8	0.317*
	ln ß	0.44±0.6	0.41±0.7	0.285
	log random	0.70±0.9	0.82±0.7	0.380*

* *p*<.05;

** *p*<.01,

*** *p*<.001.

## Discussion

The primary aim of this study was to assess for the first time one year test-retest reliability of the most challenging 4-digit and shapes versions of the CPT-IP in outpatients with schizophrenia. We found that d’ and - to a lesser degree - hit rate for correct responses revealed, only moderate long-term test-retest reliability in schizophrenia, while the same variables showed high test-retest reliability correlations in healthy controls. These high levels of long-term test retest scores in healthy controls are well in line with earlier studies. Cornblatt and colleagues (1988) evaluated test-retest reliability in 120 healthy participants over a long-term interval of 1.5 years and reported moderate to good Pearson’s correlations for d’ in digits and shapes condition [6]. Chen and Faraone (2000) reported overall good test-retest reliabilities of both d’ and hit rate in healthy participants. Both studies agreed that other CPT-IP measures generally showed less satisfactory reliabilities [13]. Three studies investigated the important methodological issue of CPT-IP test-retest reliability in patients with schizophrenia but reported reliability estimates only for short-term tests intervals. Nuechterlein and co-workers (2008) assessed 167 clinically stable patients with schizophrenia (95% retention rate) and reported very good test-retest reliability for the mean d’ value across 2-, 3-, and 4-digit conditions after 4 weeks [15]. Another large clinical trial investigated 323 clinically-stable outpatients with schizophrenia at 29 sites and confirmed good short-term test–retest reliability for the mean d’ values across 2-, 3-, and 4-digit conditions of the CPT-IP [14]. The most recent publication assessed short-term reliability on the 2-digit, 3-digit, 4-digit and shapes condition of the CPT-IP at three test time points over a total of five weeks in a similarly large sample of 43 patients with schizophrenia and 40 healthy controls. The authors demonstrated again good short-term test–retest reliability in patients with schizophrenia for d’ and hit rate for all 4 versions of the CPT-IP [16]. Overall and in contrast to our results, small differences in repeated short term test–retest reliability across three trials both for d’ and hit rate were not considered meaningfully different between groups [16].

Our study replicates previous studies which revealed high short and long-term test-retest reliability for healthy controls for the most challenging CPT-IP conditions for both d’ and hit rate. We found also consistent between-group differences for d’ and hit rate in our cross-sectional sample of healthy controls and schizophrenia patients, and thus our results replicate findings on excellent sensitivity of the CPT-IP. These results are also in line with previous findings of sustained attention deficits in individuals at risk for later development of schizophrenia-spectrum disorders and support the assertion that sustained attention impairment could be an indicator of a schizophrenia diathesis [3], [13], [23], [24].

Inclusion of cognitive measures in DSM-5 as part of diagnostic criteria for schizophrenia has been carefully considered, since cognitive impairments are relatively independent of symptom severity and stage of illness, but their discriminative value to other “boundary” disorders as bipolar and schizoaffective disorder was not considered sufficient for their inclusion in DSM-5 [25], [26]. Moreover, stability of cognitive processes varies according to assessed domains and test-retest intervals. In schizophrenia overall sufficient short term test-retest reliability was found for most cognitive measures assessing the domains: speed of processing, attention and vigilance, verbal learning, reasoning and problem solving. For other domains as working memory, visual learning and social cognition test-retest reliability varied with selected cognitive tests. For data of short term test-retest reliability for 36 included candidate cognitive tests, see Nuechterlein et al. [15].

Pietrzak et al. assessed stability of cognitive impairment in chronic schizophrenia over brief (i.e., hours) and intermediate re-test intervals (i.e., one month) using computerized tests of the domains: psychomotor function (Detection Task), visual attention/information processing (Identification task), non-verbal learning (Visual Learning Task), and executive function (Groton Maze Learning Test) and found slightly lower but good test-retest reliabilities in schizophrenia compared to healthy controls. For intermediate re-tests intervals the authors suggested that variability in cognitive performance may reflect more inherent characteristic of schizophrenia, rather than differences in test–retest reliability of cognitive measures [27].

Data on long-term neurocognitive stability in the course of illness is comparably scarce and needed in different schizophrenia patient samples, since the duration of most longitudinal studies was short to modest. A longitudinal investigation of cognitive function in schizophrenia over 1 year reported a decline in spatial recognition but not in pattern recognition or motor speed, using the Cambridge Neuropsychological Test Automated Battery (CANTAB) as part of the CUtLASS trials. In that study cognitive changes were present in schizophrenia patients but the magnitude of change was small compared with differences in cognitive measures that existed between patients [28]. A recent study assessed stability of cognition and its relation to functional outcome over a 1-year test-retest interval in 128 schizophrenia outpatients. Cognitive functioning was stable in most patients and deterioration was mainly observed for letter–number sequencing and semantic fluency tests. Estimates for 1 year test-retest reliabilities (Intra-Class Correlations, ICC), that were in our sample virtually identical to Pearsons r, varied between.50 for semantic fluency, >0.6 for recall and intrusions of the California Verbal Learning Test-II, >.7 for phonematic fluency, letter number sequencing and symbol search and up to ICC >.9 for vocabulary subtests of the Wechsler Adult Intelligence Scale–III. ICCs for functional outcome status were substantially lower within a 1 year retest interval [29].

Another recently published study assessed the course of cognitive deficits in 78 first episode schizophrenia spectrum disorder patients over a 1-year and 3-year follow-up period. Six cognitive domains assessed in this study consisted of: verbal memory (Rey Auditory Verbal Learning Test), visual memory (Rey Complex Figure Test), motor dexterity (Grooved Pegboard Test), executive functions/speed of processing (Trail Making Tests A and B), WAIS III-Backward Digits and Digit Symbol), attention and impulsivity (Continuous Performance Test- degraded-stimulus. Interestingly while first episode patients and their controls increased their performance in all cognitive measures except for verbal and visual memory, the authors identified a subgroup of 34 patients that showed a cognitive decline, associated with negative symptoms and poor functional outcome [30]. Finally, assessment of neurocognitive performance and stability in a multiplex multigenerational study of schizophrenia using a computerized neurocognitive battery calculated test-retest reliabilities (ICCs) for accuracy and speed over a 5-year interval. Compared to unaffected family members, test-retest correlations were lower in schizophrenia patients for the domains abstraction and mental flexibility; verbal memory; face memory; spatial memory; language reasoning and emotion processing. Moreover schizophrenia patients were more impaired in relation to speed than in accuracy and showed higher across-task intra-individual variability in performance compared with unaffected family members [31].

Following the aim of the current study, the incremental value of our results is constituted by adding for the first time long-term reliability data in schizophrenia for the 4-digit and shapes CPT-IP conditions. In contrast to studies using healthy controls and in contrast to studies revealing high short-term reliability in different versions of the CPT-IP in schizophrenia, we could replicate these findings only in part for a substantially longer test-retest interval and found only moderate test-retest reliability in an independent sample of outpatients with schizophrenia with mild symptom levels. Only moderate long-term reproducibility (r = .5) of d’ among patients with schizophrenia in our sample would imply that changes in CPT-IP performance may not be mainly attributed to hypothetical effects of clinical interventions, but also to random effects or noise. For clinical and practical significance and usefulness other authors proposed levels of observed agreement >.7 as a minimal necessary threshold [32], [33]. As a rule of thumb for clinical studies and in contrast to the thresholds of reliability proposed by Altman [22], Cicchetti considers reproducibility r<.7 as poor, r = .7-.79 as moderate, r = .8-.89 as good, and r>.9 as excellent [33], [34].

Our study has several strengths but also limitations: All patients included in this study were known to the authors before entering the study and had approximately monthly appointments during and after the study. Although most patients exhibited residual psychopathological symptoms, the severity was mild to moderate and psychopathology did not differ significantly between test sessions. Additionally, all patients were on stable antipsychotic medication between test session, and changes in dosages were minor and not significant. While clinical stability and insignificant changes in medication ensured that our sample is well comparable to short term studies on reliability, we believe that our study design may have influenced results on long-term stability, since an interventional study could include more clinically varying patients and thus agreement between test-sessions may even be over-estimated. On the other hand when a test is given to a very similar (homogeneous) group, the resulting scores may be too closely clustered and the reliability coefficient might therefore be actually lower than in a more heterogeneous examinee group.

In our methodological approach, only moderate test-retest stability of individual differences on CPT-IP in schizophrenia where found in spite of consistent cross-sectional differences at both sessions and in spite of clinical and medication stability during a one year interval. Additionally although our sample-size is comparable to a recent study on short term reliability of CPT-IP in schizophrenia [16], sample size is another important factor for studies of test-retest reliability. Lower test-retest reliabilities of measures would imply that larger samples are needed to detect interventional effects in clinical studies. Finally we did not control for individual factors that may influence test-retest reliability as intra-individual variability, poor motivation, fatigue, insufficient sleep, food intake or cigarette smoking prior to CPT-IP test-sessions.

In conclusion, our results imply - in contrast to most studies assessing only short-term stability of d’ in schizophrenia - at best moderate and for usage in clinical studies possibly not sufficient long-term temporal stability of d’ and hit rate for the 4-digits and the less used shapes condition of the CPT-IP. It is noteworthy that our results is limited to the 4-digit and shapes version of the CPT-IP and has been assessed in one sample of patients with schizophrenia at one site only. Further research is needed to answer the question whether less challenging versions of the CPT-IP, in different samples of patients with schizophrenia or related psychiatric disorders or a variation of intervals between test-sessions may yield better, and clinically acceptable long-term reliability of CPT-IP. In that context it is interesting that the mean across the 2, 3, and 4 digit versions of the CPT-IP, due to its high test-retest reliability in short term intervals, was chosen for the MATRICS test battery as a measure for sustained attention [14], [15].

Given our replication of consistent and stable cross-sectional differences within a one-year interval, we consider d’ – at least for short term interventional studies - also in the most challenging versions of the CPT-IP still a valuable measure for sustained attention in schizophrenia, while hit rate in both conditions could be considered a potentially valuable CPT-IP measure for the 4-digit and shapes condition. However our results of at best moderate test-retest reliability of d’ and hit-rate for a 1 year test-retest interval in our sample of clinically stable outpatients with schizophrenia may point towards less practical use of these measures when evaluating long term clinical trials to improve sustained attention in schizophrenia.
